# Engineering of Peglayted Camptothecin Into Nanomicelles and Supramolecular Hydrogels for Pesticide Combination Control

**DOI:** 10.3389/fchem.2019.00922

**Published:** 2020-01-15

**Authors:** Zhi-Jun Zhang, Xiao-Fei Shang, Liu Yang, Yan-Bin Shi, Ying-Qian Liu, Jun-Cai Li, Guan-Zhou Yang, Cheng-Jie Yang

**Affiliations:** ^1^School of Pharmacy, Lanzhou University, Lanzhou, China; ^2^Lanzhou Institute of Husbandry and Pharmaceutical Sciences, Chinese Academy of Agricultural Science, Lanzhou, China; ^3^Environmental and Municipal Engineering School, Lanzhou Jiaotong University, Lanzhou, China

**Keywords:** camptothecin, micelles, supramolecular hydrogels, combination, insecticidal activity

## Abstract

As a famous quinoline alkaloid, camptothecin (CPT) presented the significant anti-tumor activity, as well as the interesting insecticidal activities, but the low solubility, poor hydrophobicity and cuticular penetration of CPT have been severely limited the field application. In this study, we conjugated the camptothecin with polyethylene glycol, forming amphiphilic copolymer, mPEG-CPT, which could be self-assembled into micelles, or formed a hydrogel with α-CD by super-cross-linking to combine delivery with acetamiprid or nitenpyram. Results showed that the nitenpyram or acetamiprid loaded hydrogels showed dual phase release behavior, while the micelles displayed a synchronous and fast release profile. Moreover, these four nanopesticides showed potent or superior insecticidal activities and a synergetic effect against *Brevicoryne brassicae, Tetranychus cinnabarinus*, and *Bursaphelenchus xylophilus*. This finding indicated that micelles and hydrogels could be used as effective carriers for pesticide combination control.

## Introduction

Insecticides play a very important role in controlling the development of plant pests, ensuring crop productivity and promoting the steady growth of agriculture production. As a famous quinoline alkaloid and promising botanic insecticide, camptothecin (CPT) isolated from the Chinese tree *Camptotheca acuminate* (Wall et al., 1986), had been payed research attention for its interesting insecticidal activities (Hu et al., 2009; Tong et al., 2009) besides its use as an anti-tumor agent currently (Zunino et al., 2002). Camptothecin was used as a potent chemosterilant against the housefly (DeMilo and Borkovec, 1974) and cabbage caterpillar (Borkovec, 1976), and showed significant insecticidal activities against some pests including *Empoasca vitis, Nilaparvata lugens*, and *Chilo suppressalis* (Ma et al., 2010). Moreover, camptothecin showed a low toxicity to vertebrates and environment and a high insecticidal selectivity, since it mainly control insects by interfering with the reproduction potential of sexually reproducing organisms (Borkovec, 1976). However, low solubility, poor hydrophobicity and cuticular penetration have been severely hindered the field application of CPT and indicated the need for appropriate formulation and development so as to achieve improved and sustained bioavailability (Adams, 2005; Driver and Yang, 2005; Li et al., 2006).

Recently, nanotechnology represents a new impetus for sustainable agriculture development (Zhao et al., 2018), and it has been receiving increasing interest in the pesticide sector with the development of a range of nanopesticides (Khot et al., 2012; Kah et al., 2013; Melanie, 2015), since nano-pesticide formulations may offer benefits like increasing solubility and bioavailability, reducing the amount of active ingredients used and the development of resistance, as well as providing ingredient protection against premature degradation (Sasson et al., 2007; Kah et al., 2013; Kah and Hofmann, 2014). Polymeric nanospheres and nano-capsules, together with nanogels and nanofibers, even more complex nano-formulations, have been developed for the delivery of pesticides, and primarily aimed at increasing solubility or slow and controlled release profile of the active ingredients serving as protective reservoirs (Anton et al., 2008; Ao et al., 2012; Bhagat et al., 2013; Memarizadeh et al., 2014; Sharma et al., 2017). Furthermore, several nanocarriers, such as nanocapsules (Shen et al., 2010), micelles (Dong et al., 2012) and hydrogels (Ha et al., 2013) can deliver two different drugs for combination therapy. For instance, in our previous study (Ha et al., 2013), we have fabricated a multifunctional supramolecular hydrogel for loading CPT and 5-fuorouracil (5-FU).

In order to reduce the environmental pollution, increase the toxicity against pests and decrease the resistance appearance, using nanotechnology to formulate nano-based smart formulation for pesticides by virtue of nanomaterial- related properties has shown great potential for combining the different pesticides with the different modes of action. Many nanomaterials could be used as carriers for pesticide combination for controlling the development of pests. The aim of study was to conjugate the botanic pesticide camptothecin with polyethylene glycol, forming amphiphilic copolymer, mPEG-CPT. The conjugate could be self-assembled into micelles, or forming a hydrogel with α-CD by super-cross-linking to combine delivery with acetamiprid or nitenpyram. The insecticidal activities of these four nanopesticides then were evaluated against *Brevicoryne brassicae, Tetranychus cinnabarinus*, and *Bursaphelenchus xylophilus*.

## Materials and Methods

### Materials

Methoxy poly(ethylene glycol) (mPEG, MW = 350, 500, 2000) was purchased from Sigma-Aldrich without further purification. Camptothecin (CPT) was purchased from Sichuan Jiangyuan Natural Products Co. (Sichuan, China). α-Cyclodextrin (α-CD) was purchased from Aladdin Reagent Co., Ltd. (Shanghai, China). *N,N*'-Diisopropylcarbodiimide (DIC) was purchased from GL Biochem. Ltd. (Shanghai, China). All other reagents and solvents used in the study were analytical grade and obtained from commercial sources. ^1^H and ^13^C NMR were measured on a Bruker AVANCE III-400 spectrometers (Bruker Co., Karlsruhe, Germany).

### Synthesis of the mPEG-CPT Conjugates

The mPEG-CPT was synthesized according to our previously reported method (Ha et al., 2013). Briefly, to a solution of mPEG-COOH (Li et al., 2015) (1 mmol) in 10 mL of anhydrous dichloromethane was added DIC (0.17 ml, 1 mmol), 4-dimethylaminopyridine (122 mg, 1 mmol) and camptothecin (348 mg, 1 mmol) at 0 °C, and the reaction mixture was stirred for 2 h at 0°C and then allowed to warm to room temperature for 16 h. After filtration, the filtrate was washed with 0.1N HCl, dried (anhydrous MgSO_4_) and concentrated. The residue was chromatographed on a silica gel column and eluted with CHCl_3_/MeOH 20:1 to afford mPEG-CPT as a light-yellow solid.

### Preparation of the mPEG-CPT Micelles

The mPEG-CPT micelles were prepared by dialysis method. Briefly, to a solution of mPEG-CPT (10 mg) in 1.0 mL DMSO was added dropwise 10 mL deionized water at room temperature. The resulting solution was stirred overnight. Then the solution was loaded into a dialysis bag (MWCO 3500) and dialyzed against 3 L deionized water for 2 days.

### Preparation of Acetamiprid or Nitenpyram-Loaded mPEG_2000_-CPT Micelles

mPEG_2000_-CPT (300 mg) was dissolved in 2 mL DMSO, 200 mL deionized water and 1 mL DMSO solution containing 10 mg acetamiprid (or nitenpyram) were added successively into the solution under stirring at room temperature. After stirring overnight, the solution was loaded into a dialysis bag (MWCO 3500), dialyzed against 10 L deionized water for 2 days.

The mPEG-CPT micelles were prepared by dialysis method. Briefly, mPEG-CPT (10 mg) was dissolved in 1.0 mL of DMSO, then 10 mL deionized water was added into solution dropwise under stirring at room temperature. The resulting solution was stirred overnight. Then the solution was loaded into a dialysis bag (MWCO 3500) and dialyzed against 3 L deionized water for 2 days.

### Formation of Acetamiprid- or Nitenpyram-Loaded mPEG_2000_-CPT-α-CD Hydrogels

To An aqueous solution of α-CD (100 mg/mL) and 50 mg acetamiprid (or nitenpyram) was added an aqueous solution of mPEG_2000_-CPT (60 mg/mL). For all samples, the solution was mixed thoroughly by sonication for 5 min followed by incubation at room temperature for 72 h before measurements.

### Characterization

#### Self-Aggregation Behavior of mPEG-CPT Conjugates

The mPEG-CPT conjugates suspension was prepared in the same way as the micelle preparation, and the micelle solution was adjusted to various concentrations (from 0.00001 to 5 mg/mL), a known amount of pyrene in methanol was evaporated at 40°C. A total of 3 mL of various concentration of sample suspension was added to each vial, and heated for 3 h at 65°C to equilibrate the pyrene and the nanoparticles, and remained undisturbed to cool overnight at room temperature. The critical micelle concentration (CMC) was determined as previous work (Ha et al., 2013). Pyrene was used as a fluorescence probe. The final concentration of pyrene was fixed at 1.0 μM. Fluorescent spectra were measured using fluorescence spectrophotometer (RF-5301PC, SHIMADZU, Japan) with a slit width of 10.0 and 2.5 nm for excitation and emission. For fluorescence excitation spectra, the emission wavelength was set at 390 nm.

#### The Size and Size Distributions of the Micelles

The size and size distributions of the micelles in aqueous solutions were determined by dynamic light scattering (DLS) using a laser light scattering spectrometer (BI-200SM, Brookhaven, USA) at the wavelength 514 nm; the scattering angle is 90°. Then, after dropping onto the carbon-coated 300 mesh copper grid from samples solution, the grids were air-dried at room temperature. The morphology and size of conjugates was observed by a JEM-1200EX transmission electron microscopy (JEOL, Japan).

#### Drug Loading Content and Encapsulation Efficiency

The drug-loaded micelles solution and the blank micelles solutions were prepared at the same way. The amount of acetamiprid (or nitenpyram) was detected by UV spectrometry at 270 nm (or 246 nm) and calculated with standard curve plotted by a stand acetamiprid (or nitenpyram) solution. Drug loading content and drug encapsulation efficiency were calculated according to the following equation:

$$\begin{array}{l} \text{ Drug loading content}\left(\text{\%}\right)=\frac{{\text{W}}_{\text{drug in nanocapsules}}}{{\text{W}}_{\text{nanocapsules}}}\times 100\\ \text{Encapsulation efficiency}\left(\text{\%}\right)=\frac{{\text{W}}_{\text{drug in nanocapsules}}}{{\text{W}}_{\text{feeding drug}}}\times 100 \end{array}$$

#### Scanning Electron Microscopy

For the SEM observations, the specimens were freeze-dried under vacuum and ground to fine powder. The powder was placed on conducting glue and coated with gold vapor and then analyzed on a JSM-5600LV electron microscope (JEOL, Japan). All tests were performed at 25 °C.

### *In vitro* Release Kinetics Studies of Hydrogels and Micelles

100 mg α-CD and 10 mg acetamiprid (or nitenpyram) was added into 1.0 mL mPEG2000-CPT conjugate solution (24.0 mg/mL), and the solution was added into a 1.5 mL cuvette. Then, the solution was mixed thoroughly by sonication for 5 min followed by incubation at 37°C for 72 h, allowing the mixture to form a viscous hydrogel. The cuvette was placed upside-down in a test tube with 30.0 mL of deionized water and incubated at room temperature. The concentrations of the acetamiprid (or nitenpyram) and mPEG2000-CPT released from hydrogels were determined using an Agilent 1100 high performance liquid chromatography (HPLC). Chromatographic separation was performed on an Eclipse Plus C18 column (4.6 × 250 mm, 5 μm) at 25°C with methanol and 0.1% phosphoric acid aqueous solutions (75:25, v/v) as mobile phase at a flow rate of 1.0 mL/min. A wavelength of 372 nm was used to detect mPEG2000-CPT, and 270 nm to nitenpyram (246 nm to acetamiprid). The concentrations of mPEG2000-CPT and nitenpyram (or acetamiprid) were calculated based on the equation for calibration curve.

The release of CPT and acetamiprid (or nitenpyram) from the mPEG_2000_-CPT micelles was analyzed by a dialysis method, and mPEG_2000_-CPT solution (1.0 mL) at deionized water (mPEG_2000_-CPT at 20.0 mg/mL) was loaded into a dialysis bag (MWCO 3500). The dialysis bag was then immersed in 30.0 mL of deionized water at room temperature. Deionized water medium (3.0 mL) was withdrawn at timed intervals and replaced by 3.0 mL fresh deionized water to maintain submersed conditions. The concentrations of CPT and acetamiprid (or nitenpyram) were determined by HPLC.

### Insecticidal Activity Assay

To evaluate the insecticidal efficacy of the obtained micelles and hydrogels, three insects *B. brassicae, T. cinnabarinus*, and *B. xylophilus* were used in this test. The adults of *B. brassicae* were collected from suburban fields of Lanzhou, Gansu Province, China, and reared in our laboratory under controlled photoperiod (12:12 h light: dark) and temperature (25 ± 1°C). The female adults of *T. cinnabarinus* were obtained from Zhejiang Chemical Industry Research Institute, reared on Horsebean seedling, and maintained in incubators at 26 ± 1°C, 70 ± 5% relative humidity, with a photoperiod of 14/10 h light-dark cycle. *B. xylophilus* was isolated by the Baermann method (Viglierchio and Schmitt, 1983) from chips of an infected masson pine collected from Fuyang area, Zhejiang Province, China. *B. xylophilus* was reared on a lawn of *Botrytis cinerea* that was cultured on potato dextrose agar (PDA) plates at 25°C and separated using the Baermann method. The separated *B. xylophilus* was washed with sterile water to remove the surface bacterial and fungal contaminants. The insecticidal activities of these four nanopesticides against *B. brassicae, T. cinnabarinus*, and *B. xylophilus* were evaluated according to the reported procedure (Nagase et al., 2006; Wang et al., 2006; Zhao et al., 2010). All treatments were triplicated and incubated at 25°C for 24 h, and control groups were tested with acetone only.

### Statistical Analysis

Results were expressed as the mean ± standard deviation (SD) and probit analysis was used to determine lethal concentrations of 50% (LC_50_) using the SPSS program (version 13.0).

After determining the LC_50_ for each combination, a co-toxicity coefficient (CTC) (Sun and Johnson, 1960) for mixed formulation experiments were then calculated according to the following: if CTC>120, it shows a synergistic effect, whereas CTC <80 indicates an antagonistic effect, and CTC between 80 and 120 is considered as an additive effect. If a mixture (M) compounds of two parts (A and B), and both components have LC_50_, then the following formulas are used (A serving as the standard):

$$\begin{array}{l} \text{ Toxicity index }\left(\text{TI}\right)\text{ of A}=100\\ \text{ Toxicity index }\left(\text{TI}\right)\text{ of B}=\frac{\text{L}{\text{C}}_{50}\;of\;A}{\text{L}{\text{C}}_{50}\;of\;B}\times 100\\ \text{ Actual Toxicity index }\left(\text{ATI}\right)\text{ of M}=\frac{\text{L}{\text{C}}_{50}\;of\;A}{\text{L}{\text{C}}_{50}\;of\;M}\times 100\\ \text{Theoretical Toxicity index}\left(\text{TTI}\right)\text{ of M}=\text{TI of A}\times \text{\% of A in M}\\ \;+\text{TI of B}\times \text{\% of B in M}\\ \text{ Cotoxicity coefficient }\left(\text{CTC}\right)=\frac{\text{ATI of M}}{\text{TTI of M}}\times 100 \end{array}$$

## Results and Discussion

### Synthesis of mPEG-CPT Derivatives

**Figure 1:**
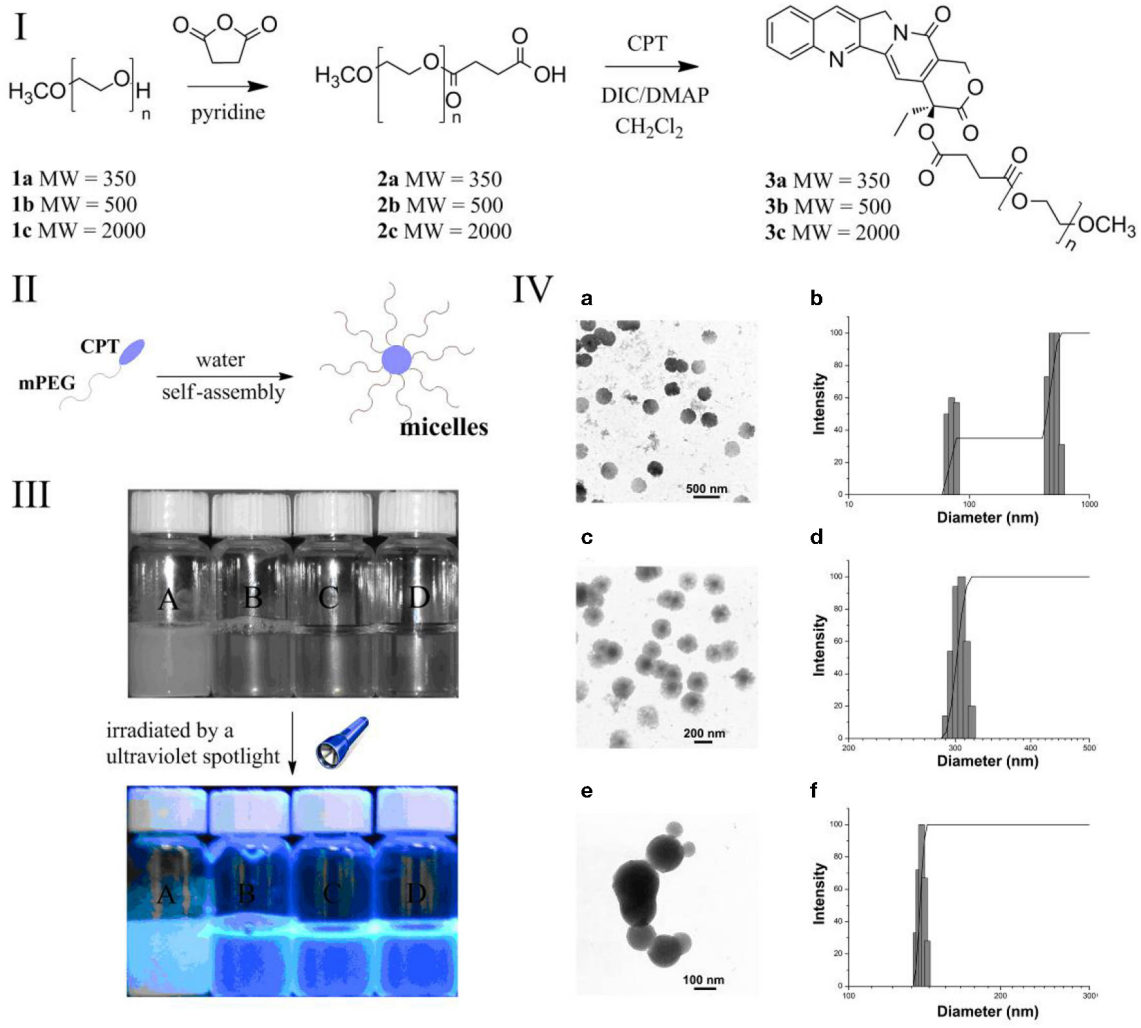
**(I)** Synthesis of mPEG-CPT derivatives; **(II)**: optical photo of (A) CPT (0.5 mg/mL), the micelles formed by (B) mPEG350-CPT (0.5 mg/mL), (C) mPEG500-CPT (1 mg/mL) and (d) mPEG2000-CPT (10 mg/mL), and all samples observed under the ultraviolet spotlight at the wavelength of 365 nm; **(III)**: schematic of the micelles formed by mPEG-CPT derivatives; **(IV)** mPEG350-CPT-formed micelles observed by transmission electron microscopy (TEM) (a) and the micelles measured by dynamic laser light scattering (DLS) (b); mPEG500-CPT-formed micelles observed by TEM (c) and the micelles measured by DLS; mPEG2000-CPT-formed micelles observed by TEM (e) and the micelles measured by DLS (f).

The synthesis of mPEG-CPT derivatives was shown in Figure 1I. A class of low-molecular-weight (MW) methoxypolyethylene glycols (mPEG) (Mn = 350, 500 and 2000) were used. The mPEG chain with terminal carboxylic acids was first synthesized via succinic anhydride, and then reacted with CPT molecules to produce the mPEG-CPT under DIC/DMAP condition. The structure of the resulting mPEG-CPT derivatives was confirmed by ^1^H-NMR, ^13^C-NMR and FT-IR (data in supporting Information). After the modification by low-MW mPEG, the mPEG-CPT conjugates had a better water solubility than the free CPT (about 3 μg/mL) (Shen et al., 2010). Along with the increase of chain length of mPEG, the water solubility was increased (Figure 1III). For example, the water solubility of mPEG_2000_-CPT was above 60 mg/mL (> 7.2 mg/mL in terms of CPT), which was 2×10^3^ times higher than that of the free CPT.

### Formation and Characterization of mPEG-CPT Micelles

In an amphiphilic block copolymer, the hydrophobic and hydrophilic parts are connected to balance the amphiphilicity for forming micelles (Figure 1II). The fluorescence probe technique was applied to study the self-aggregation behavior of mPEG-CPT conjugates on a molecular level. Pyrene was chosen as the fluorescent probe due to its photo-physical properties. Figure S1 demonstrates the intensity ratio (*I*_337_/*I*_332_) of the pyrene excitation spectra vs. the logarithm of the concentration of mPEG-CPT. The critical aggregation concentration (CAC) values of the mPEG-CPT (MW = 350, 500 and 2000) conjugates are in the range of 5.62 × 10^−4^ to 3.16 × 10^−3^ mg/mL, which are one order of magnitude lower than those of low molecular weight sufactants, and even more lower than those of other polymeric amphiphiles. The lower molecular weight of PEG chain induces the lower CAC. The morphology of mPEG-CPT self-aggregates was investigated by TEM (Figure 1IV), from the TEM images, an obvious contrast between the central and outer part of particle was observed, which is typical of micelle as reported for different kinds of polymeric micelles. The size of the micelles in aqueous solution measured by dynamic laser light scattering (DLS) are in the range from 139.5 to 350.2 nm, which were obviously larger than the sizes determined by TEM. These results indicated that during the process of the solvent evaporation in the samples preparation the particles were contracted. Furthermore, the DLS results indicated that the longer chain of mPEG is favorable to form smaller and uniform micelles, thus, mPEG_2000_-CPT micelles was adopted as a carrier of pesticides.

**Figure 2:**
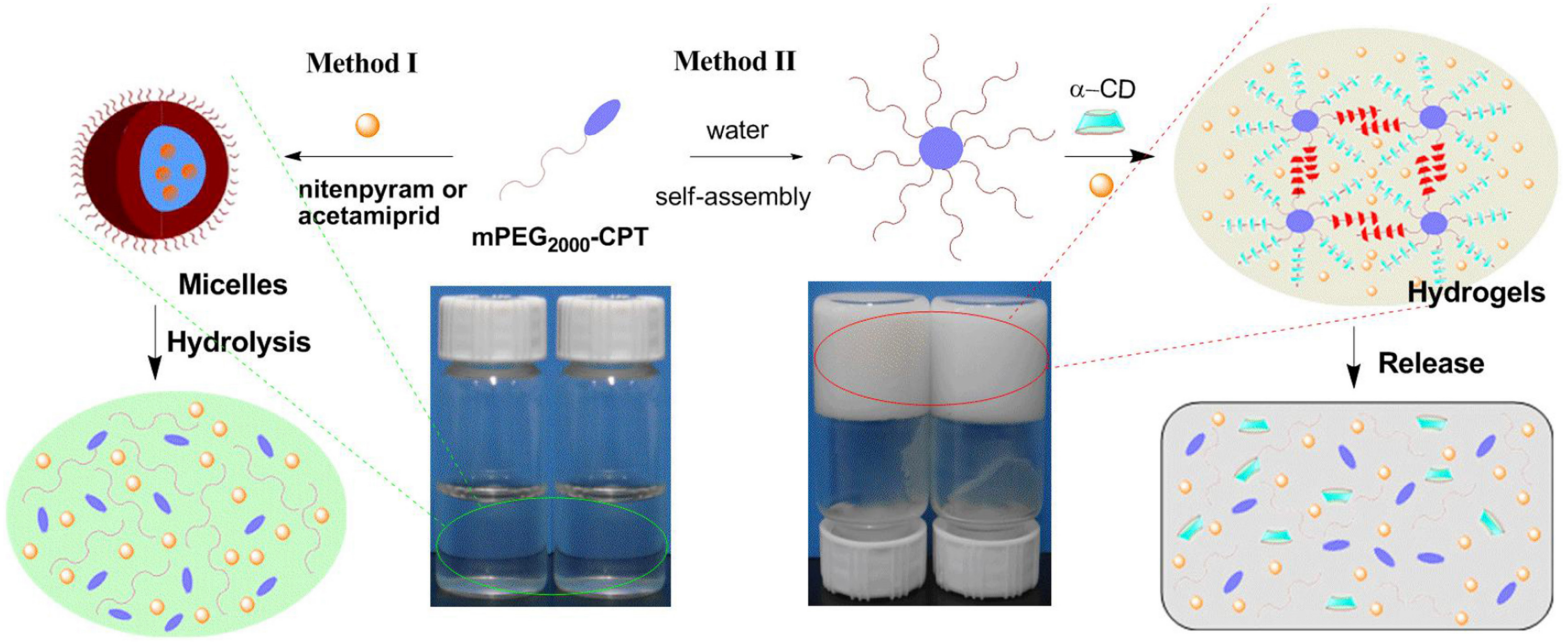
Schematic of the micelles formed by mPEG2000-CPT after loading with nitenpyram (or acetamiprid) (Method I) and the supramolecular hydrogels made of mPEG2000-CPT (24 mg/mL) with nitenpyram (10 mg/mL) or acetamiprid (10 mg/mL) (Method II). For the last two samples, [α-CD] =100 (mg/mL).

**Figure 3:**
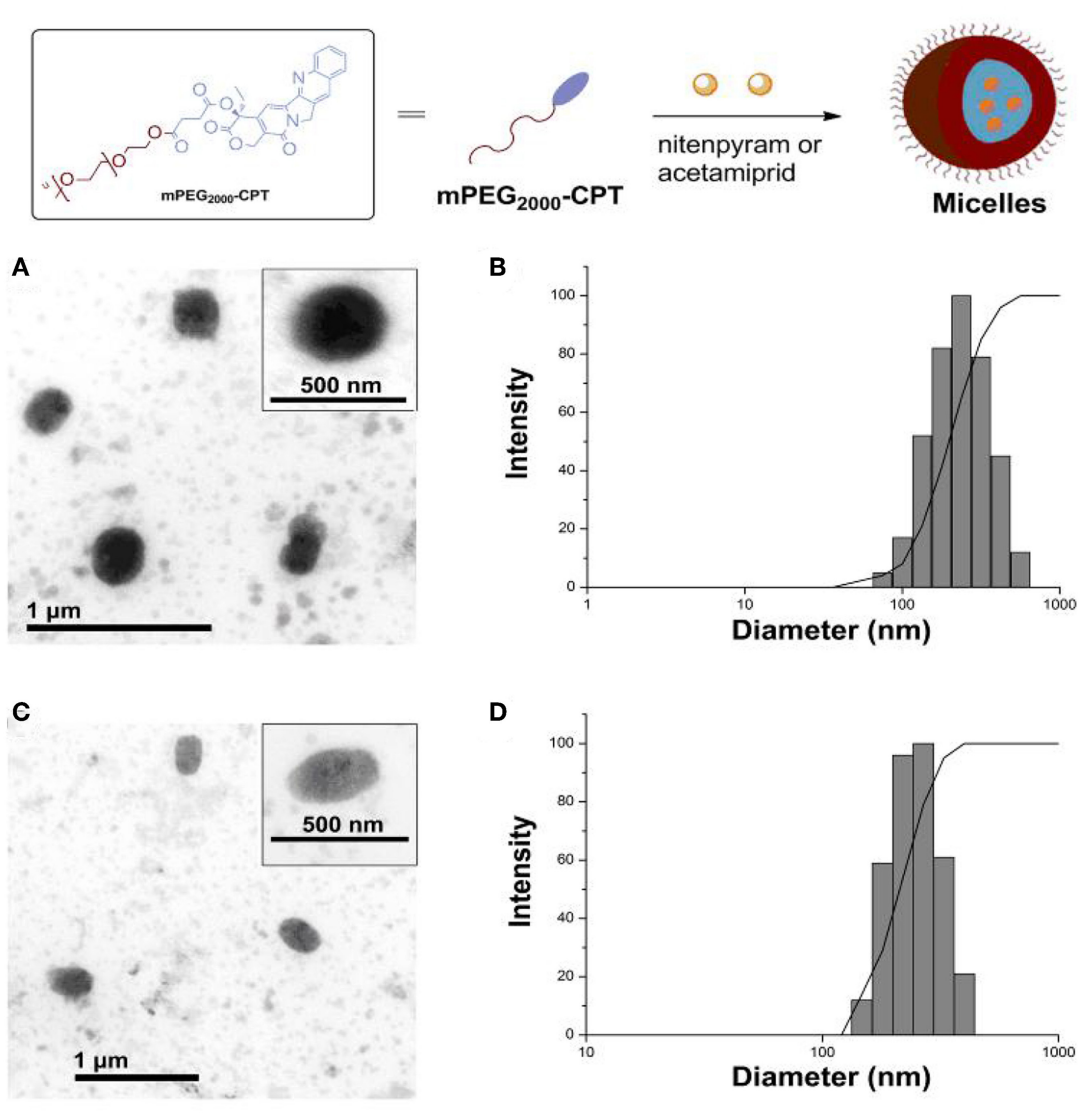
(Top) Schematic of the micelles formed by mPEG_2000_-CPT after loading with nitenpyram (or acetamiprid). (Bottom) mPEG_2000_-CPT-formed micelles observed by transmission electron microscopy (TEM) **(A)** and measured by dynamic laser light scattering (DLS) **(B)** after loading with acetamiprid; mPEG_2000_-CPT-formed micelles observed by TEM **(C)** and measured by DLS **(D)** after loading with nitenpyram.

The structures of mPEG_2000_-CPT micelles were further confirmed by encapsulation behavior for water-soluble insecticide nitenpyram and poor water-soluble insecticide acetamiprid (Figure 2). Nitenpyram (or acetamiprid) was loaded into the micelles using a dialysis method. The incorporation of acetamiprid into mPEG_2000_-CPT micelles occurred simultaneously during dialysis and the encapsulation efficiency achieved to 20.69%. However, the encapsulation efficiency of nitenpyram was 13.90%, which was slightly lower than that of acetamiprid. Thus, loading of poor water-soluble compounds into the nanoparticles further confirmed their micelle structure. The acetamiprid or nitenpyram loaded micelles became slightly larger, but more uniform in size (Figure 3). For instance, mPEG_2000_-CPT/acetamiprid micelles were about 387.2 nm in diameter, and the mPEG_2000_-CPT/Nitenpyram micelles became 350.6 nm. The increase of the micelle sizes after loaded with pesticides may cause by the insertion of the hydrophobic part of pesticides into the core of micelles.

### Formation and Characterization of Hydrogels

**Figure 4:**
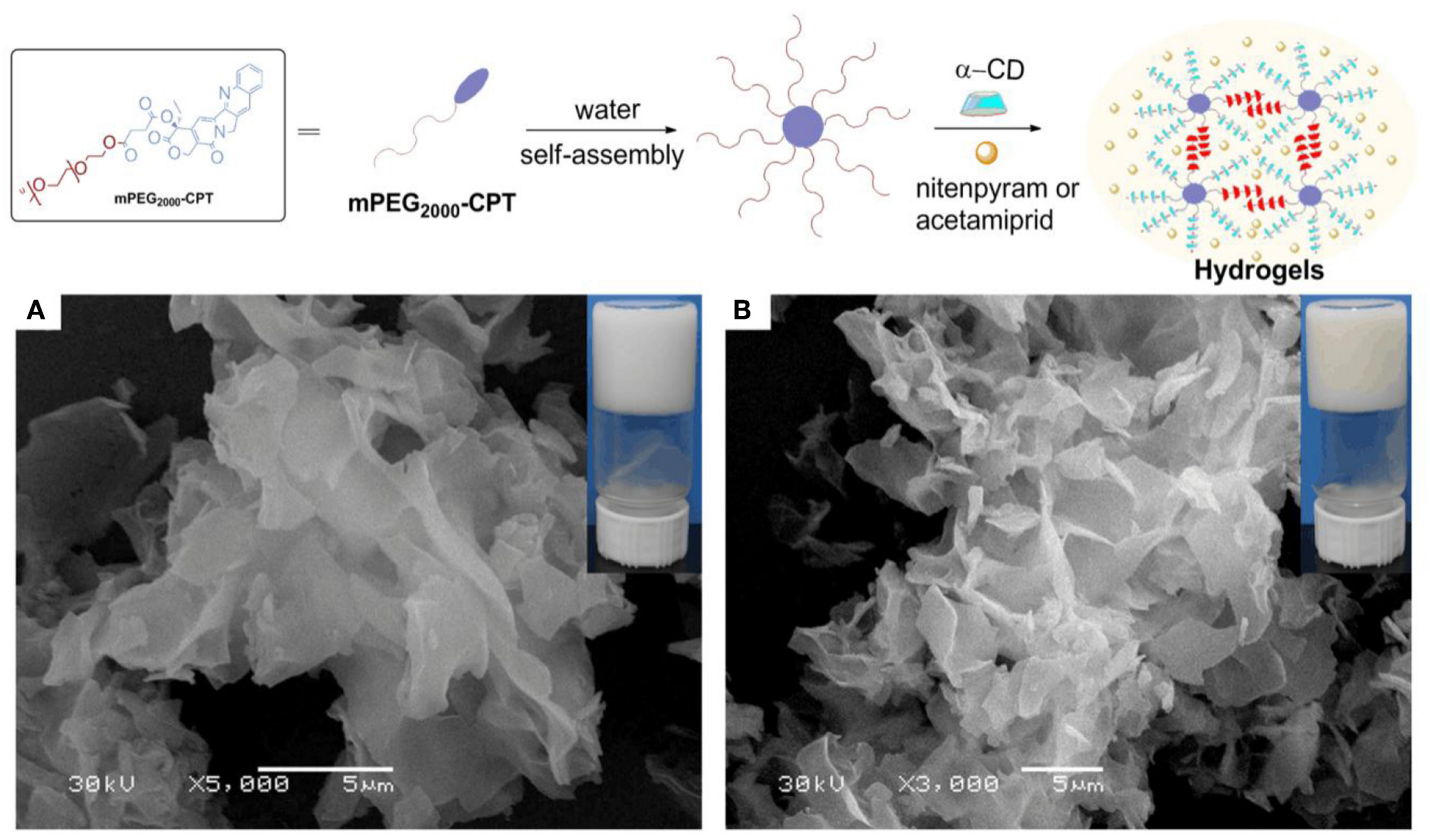
(Top) Schematic of the supramolecular hydrogels made of mPEG_2000_-CPT with nitenpyram or acetamiprid. (Bottom) SEM images of mPEG_2000_-CPT/α-CD-formed hydrogels including different pesticides, acetamiprid **(A)**, or nitenpyram **(B)**. For all samples, [mPEG_2000_-CPT] = 24 (mg/mL), [pesticides] = 10 (mg/mL) and [α-CD] =100 (mg/mL).

The loading content of acetamiprid or nitenpyram in mPEG_2000_-CPT micelles was extremely low. Thus, we devoted to find a new carrier to improve loading content of acetamiprid or nitenpyram. In our previous study (Ha et al., 2013), we have successfully used the hydrophobic drug CPT as a building block for constructing supramolecular hydrogels for combination therapy with 5-FU. Herein, in this work, we try to load acetamiprid or nitenpyram into such multifunctional supramolecular hydrogel for combination use. The schematic formation process of such hydrogels is shown in Figure 2 (Method II) and Figure 4. The mPEG_2000_-CPT conjugates forming hydrogels based on supra-cross-links between one end of mPEG_2000_ blocks and α-CD as well as the hydrophobic aggregation of the CPT groups. Furthermore, due to the porous structure and shear-thinning properties, the resulting hydrogels could be loaded with nitenpyram and acetamiprid during the formation of hydrogels. Experimental results showed that the encapsulation efficiency and loading content of acetamiprid or nitenpyram in hydrogels have improved significantly which are 100% and 7.45 wt% respectively, compared to those in micelles which are 20.69%, 1.125 wt% and 13.90%, 0.796 wt%, respectively.

The morphology of hydrogels was investigated by SEM (Figures 4A,B). In our previous work (Ha et al., 2013), we find the mPEG_2000_-CPT/α-CD-formed hydrogel clearly demonstrate the presence of a typical porous structure. Obviously, it shows the obtained hydrogels had a petal structure after loading acetamiprid or nitenpyram. We believe this may result from the network structure of the formed hydrogels was fill with molecules of acetamiprid or nitenpyram during the process of encapsulation.

### Release Profiles of mPEG_2000_-CPT Micelles and Hydrogels

**Figure 5:**
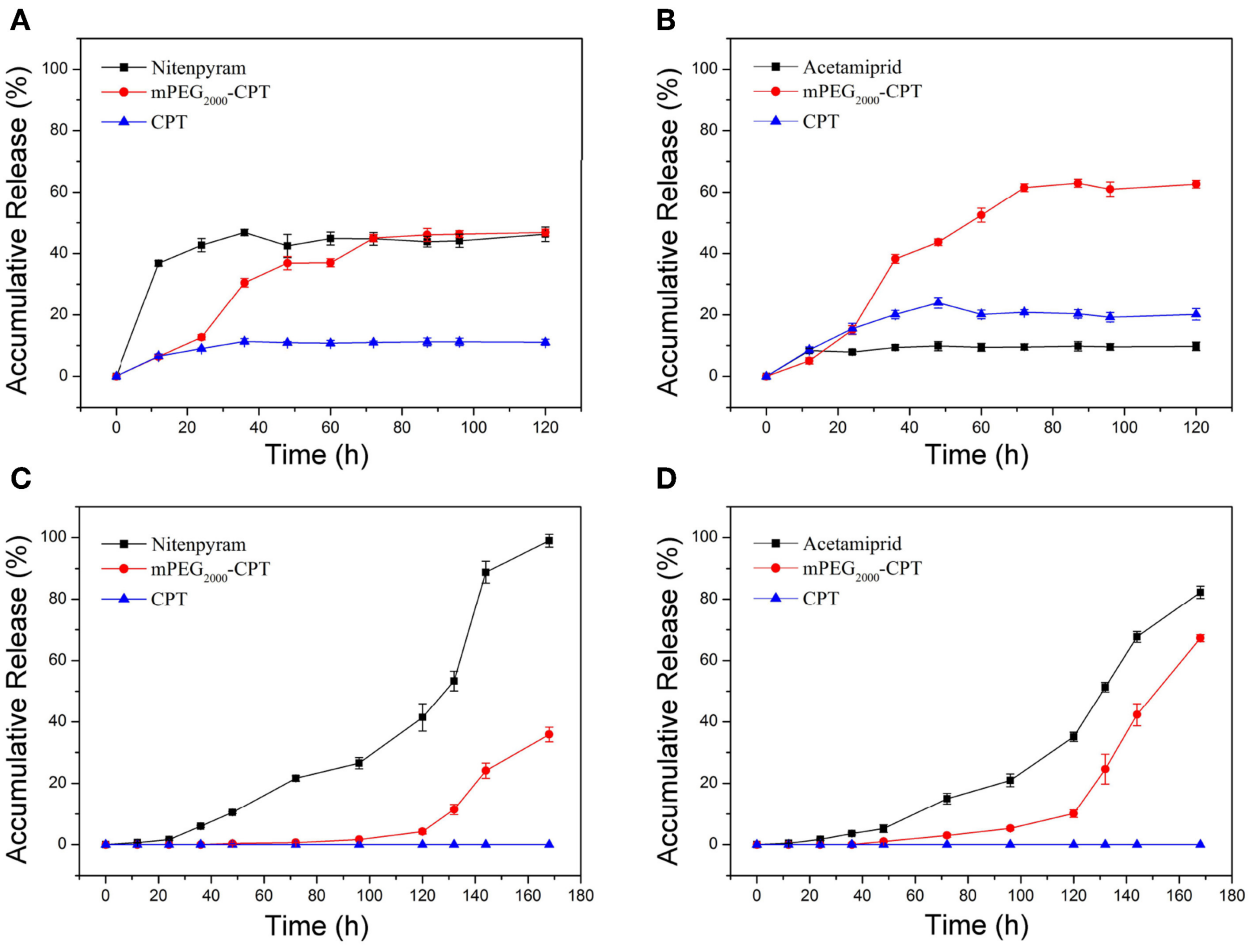
Release profiles of CPT, mPEG_2000_-CPT and nitenpyram (or acetamiprid) from mPEG_2000_-CPT/α-CD hydrogels loaded with nitenpyram **(A)** and acetamiprid **(B)**, and mPEG_2000_-CPT nanoparticles loaded with nitenpyram **(C)** and acetamiprid **(D)** in deionized water at room temperature.

The release profiles of mPEG_2000_-CPT micelles and hydrogels loaded with nitenpyram or acetamiprid were showed in Figure 5. Sustaining the release of nitenpyram or acetamiprid for more than 168 h accompanied with some release of mPEG_2000_-CPT, and none release of CPT was observed, indicating that the mPEG_2000_-CPT hydrogels had a dual-phase releasing behavior (Figures 5A,B). Similar results were seen in our previous study (Ha et al., 2013). Nitenpyram or acetamiprid was released from the hydrogels mainly by the diffusion and the breakup of the supra-cross-links of mPEG_2000_-CPT-α-CD in the first stage. The partial framework of hydrogel was gradually dissociated along with the release of mPEG-CPT and nitenpyram or acetamiprid. In the second phase, CPT is released by mPEG2000-CPT hydrolysis in the presence of esterase which is abundant in cytoplasm. However, a synchronous release of CPT, mPEG_2000_-CPT and nitenpyram (or acetamiprid) from the micelles could be observed. mPEG_2000_-CPT was hydrolyzed very slowly and released CPT and nitenpyram (or acetamiprid) in deionized water without a burst release phenomenon (Figures 5C,D). Similarly, it would be quickly hydrolyzed and release CPT and nitenpyram (or acetamiprid) in the presence of esterase.

### Biological Activity

**Figure 6:**
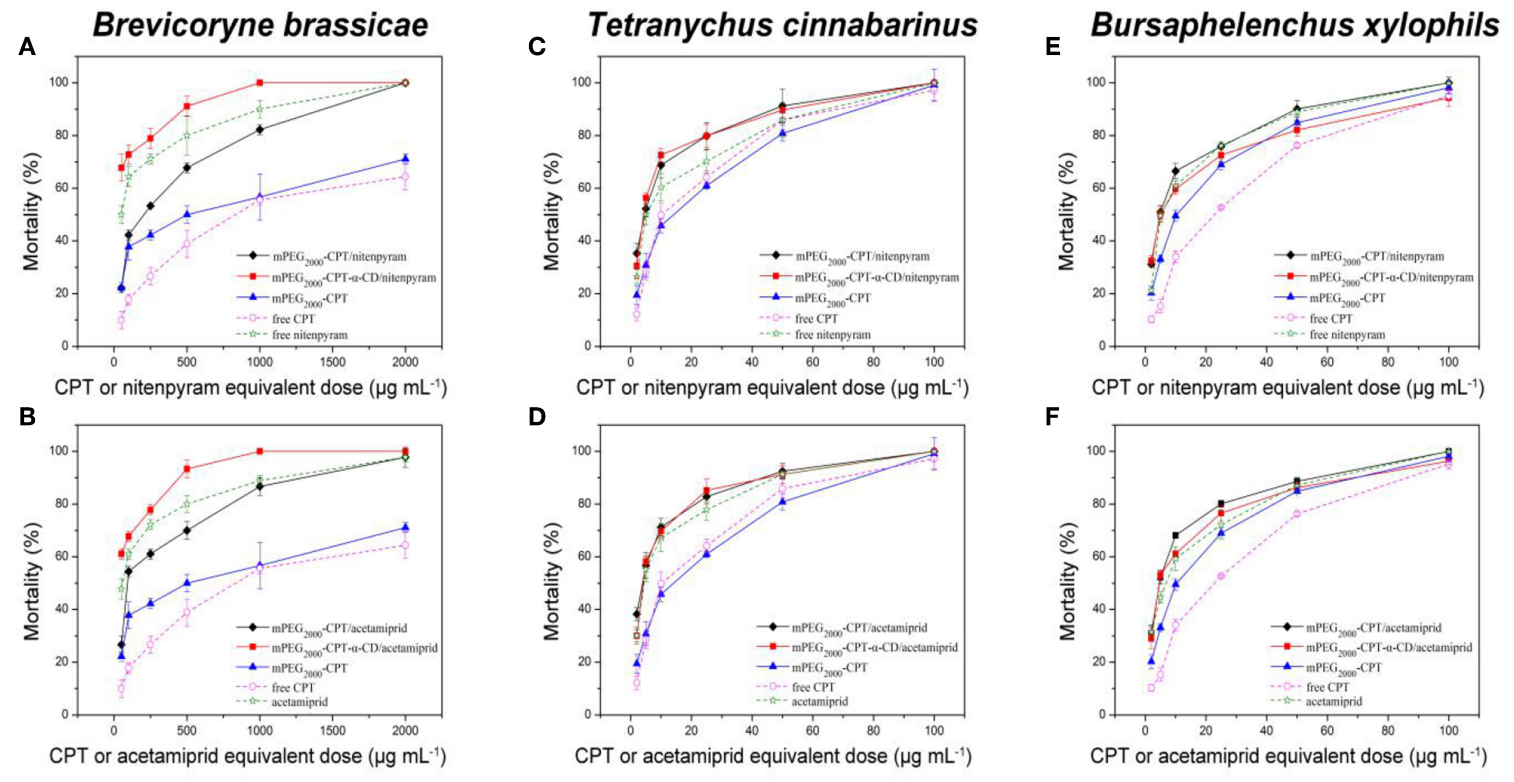
Mortality of *B. brassicae, T. cinnabarinus*, and *B. xylophils* treated with mPEG_2000_-CPT/niterpyram, mPEG_2000_-CPT-α-CD/nitenpyram **(A,C,E)**, mPEG_2000_-CPT/acetamiprid and mPEG_2000_-CPT-α-CD/acetamiprid **(B,D,F)**. Data were given as mean ±SD (*n* = 3).

The insecticidal activities of mPEG_2000_-CPT micelles and hydrogels loaded with nitenpyram and acetamiprid against *B. brassicae, T. cinnabarinus*, and *B. xylophilus* were evaluated, respectively. From Figure 6 and Table 1, we can see that these four nanopesticides, mPEG_2000_-CPT micelles and hydrogels loaded with nitenpyram and acetamiprid, all exhibited good to excellent insecticidal activities against *B. brassicae, T. cinnabarinus*, and *B. xylophilus*, with LC_50_ values ranging from 29.17 to 176.72 μg/mL, 3.59 to 4.27 μg/mL and 4.70 to 5.56 μg/mL, which were more potent than those of CPT. Among of them, mPEG_2000_-CPT hydrogels loaded with nitenpyram or acetamiprid were found to be equally potent or superior insecticidal activities against three pests to their single agent nitenpyram or acetamiprid. When *T. cinnabarinus* and *B. xylophilus* were fed with the diet containing 100 μg/mL, the corrected mortality rates of two mPEG_2000_-CPT hydrogels had 100% inhibitory effect. At 25 μg/mL, they still remaining had excellent inhibitory activity and had >75% mortality. Even at 5 μg/mL in diet, they also exhibited good insecticidal activities against three pests. Similar results were observed after treatment with mPEG_2000_-CPT micelles loaded with nitenpyram or acetamiprid against *T. cinnabarinus* and *B. xylophilus*. However, mPEG_2000_-CPT micelles loaded with nitenpyram or acetamiprid displayed the lower insecticidal activity than nitenpyram or acetamiprid alone against *B. brassicae*. Furthermore, mPEG_2000_-CPT micelles loaded with nitenpyram or acetamiprid showed lower LC_50_ values than the corresponding hydrogels against *T. cinnabarinus* and *B. xylophilus*, while the opposite result was obtained against *B. brassicae*. We thought that the higher activity of mPEG_2000_-CPT micelles loaded with nitenpyram or acetamiprid against *T. cinnabarinus* and *B. xylophilus* is that the synchronous and fast release profiles of CPT, mPEG_2000_-CPT and nitenpyram (or acetamiprid) from the micelles may lead to quickly kill these small bugs.

**Table 1:** LC_50_-probit values and co-toxicity coefficient for the contact mortality of *B. brassicae, T. cinnabarinus*, and *B. xylophils* treated with these four nanopesticides after 24 h of exposure.

**Samples**	***B. brassicae***		***T. cinnabarinus***		***B. xylophils***	
	**LC_**50**_ (μg/mL)**	**CTC**	**LC_**50**_ (μg/mL)**	**CTC**	**LC_**50**_ (μg/mL)**	**CTC**
mPEG_350_-CPT	551.58 ± 10.94^*^	-	10.55 ± 3.33	-	13.32 ± 1.76	-
mPEG_500_-CPT	572.33 ± 18.35^*^	-	10.28 ± 1.92	-	12.72 ± 1.19	-
mPEG_2000_-CPT	430.47 ± 9.67^*^	-	11.83 ± 1.92	-	9.61 ± 0.67^*^	-
mPEG_2000_-CPT /nitenpyram	176.72 ± 5.91^**^	206.77	4.26 ± 0.67^**^	251.21	4.97 ± 0.33^**^	216.31
mPEG_2000_-CPT /acetamiprid	126.01 ± 8.76^**^	322.93	3.59 ± 0.33^**^	273.50	4.70 ± 0.67^**^	220.69
mPEG_2000_-CPT-α-CD/nitenpyram	29.17 ± 3.33^**^	207.41	4.27 ± 0.89^**^	159.67	5.56 ± 0.23^**^	126.28
mPEG_2000_-CPT-α-CD /acetamiprid	36.02 ± 5.67^**^	266.69	4.22 ± 1.21^**^	123.93	5.37 ± 0.76^**^	122.42
CPT	852.07 ± 26.67	-	11.44 ± 1.92	-	11.44 ± 1.34	-
nitenpyram	50.72 ± 6.93	-	6.29 ± 0.33	-	6.50 ± 1.23	-
acetamiprid	81.12 ± 4.67	-	4.70 ± 1.17	-	6.04 ± 1.19	-

* *P < 0.05 compared to the CPT-treated group*.

** *P < 0.01 compared to the CPT-treated group*.

### Combined Toxicity of mPEG_2000_-CPT Micelles and Hydrogels

The effectiveness of mPEG_2000_-CPT micelles and hydrogels against *B. brassicae, T. cinnabarinus* and *B. xylophilus* increased significantly, compared to camptothecin. To evaluate the joint action of CPT and acetamiprid (or nitenpyram), Sun's co-toxicity coefficient values (CTC) were used (Sun and Johnson, 1960). Calculation of the CTC values for mPEG_2000_-CPT micelles and hydrogels indicated that there was a synergistic effect between CPT and acetamiprid (or nitenpyram), with CTC values ranging from 122.42 to 322.93 (Table 1).

Interestingly, the CTC values of mPEG_2000_-CPT micelles against three insets were larger than that of hydrogels after loading with acetamiprid, 322.93 and 266.69 for *B. brassicae*; 273.50 and 123.93 for *T. cinnabarinus*; 220.69 and 122.42 for *B. xylophilus*, respectively. Similarly, the CTC values of mPEG_2000_-CPT/nitenpyram micelles against three insets were larger than those of mPEG_2000_-CPT-α-CD/nitenpyram hydrogels, 206.77 and 207.41 for *B. brassicae*; 251.21 and 159.67 for *T. cinnabarinus*; 216.31 and 126.28 for *B. xylophilus*, respectively, which were in agreement with the toxicity levels.

summary, we used micelles and supramolecular hydrogels as carriers for loading CPT and nitenpyram or acetamiprid for pesticide combination control. The hydrophobic pesticide CPT was conjugated to a low-MW mPEG, forming amphiphilic conjugates mPEG-CPT. The conjugates formed stable micelles which could be loaded with nitenpyram or acetamiprid. Meanwhile, the conjugates mPEG-CPT formed stable hydrogels based on supra-cross-links between one end of mPEG_2000_ blocks and α-CD as well as the hydrophobic aggregation of the CPT groups, and loaded with nitenpyram and acetamiprid during the formation of hydrogels. The nitenpyram and acetamiprid loaded hydrogels showed dual phase release behavior, while the micelles display a synchronous and fast release profile. The bioassay results showed that these four nanopesticides exhibited potent or superior insecticidal activities and a synergetic effect to three pests, *B. brassicae, T. cinnabarinus*, and *B. xylophilus*. This finding may provide two novel, efficient and mild approaches for the development of micelles and hydrogels as novel materials in pesticide combination control.

## Data Availability Statement

All datasets generated for this study are included in the article/Supplementary Material.

## Author Contributions

Z-JZ and Y-QL conceived and designed the study. Z-JZ and Y-BS established the experimental protocols. X-FS and LY contributed analytical tools. Z-JZ, G-ZY, J-CL, and C-JY performed the experiments and analyzed the data. Z-JZ wrote the manuscript. All authors read and approved the manuscript.

### Conflict of Interest

The authors declare that the research was conducted in the absence of any commercial or financial relationships that could be construed as a potential conflict of interest.
