# Supplementary material for: Engineering of Peglayted Camptothecin Into Nanomicelles and Supramolecular Hydrogels for Pesticide Combination Control
Source: Front Chem. 2020 Jan 15;7:922. doi: 10.3389/fchem.2019.00922 (PMC6974551; doi:10.3389/fchem.2019.00922)
Supplement: Supplementary file 1 [file Table_1.DOC]

**Supplementary material for**

**Engineering of Peglayted Camptothecin into Micelles and Supramolecular Hydrogels for Pesticide Combination Control**

Zhi-Jun Zhang^1^, Xiao-Fei Shang^1,3^, Liu Yang^2^, Yan-Bin Shi^1^, Ying-Qian Liu^1*^, Jun-Cai Li^1^, Guan-Zhou Yang^1^, Cheng-Jie Yang^1^

^1^ School of Pharmacy, Lanzhou University, Lanzhou 730000, P.R. China

^2^ Environmental and Municipal Engineering School, Lanzhou Jiaotong University, Lanzhou 730000, PR China

^3^ Lanzhou Institute of Husbandry and Pharmaceutical Sciences, Chinese Academy of Agricultural Science, Lanzhou 730050, P.R. China.

*Corresponding author: Ying-Qian Liu,

Tel. +86(0)931-8618795 E-mail [yqliu@lzu.edu.cn](mailto:yqliu@lzu.edu.cn)

**Chemistry**

**Preparation of the mPEG-CPT** **derivatives**

mPEG (Mn = 350, 500 and 2000, 2 mmol) and succinic anhydride (1 g, 10 mmol) (dried over P_2_O_5_ and in high vacuum) were dissolved in 25 mL anhydrous dichloromethane (weight-volume ratio 20%) and refluxed for 3 days under nitrogen with pyridine (0.8 mL, 10 mmol) as catalyst. After evaporation of the solvent, the crude product was dissolved in water. Unreacted succinic anhydride was eliminated by washing with a mixture of ethyl acetate and hexane (v:v = 1:1) for several times. The aqueous phase was extracted by CHCl_3_, and all organic layers were merged followed by drying over anhydrous MgSO_4_ overnight. The filtrate was concentrated and recrystallized from diethyl ether to give carboxyl-functionalized mPEG (mPEG-COOH).

mPEG-COOH (1 mmol) was dissolved in 10 mL of anhydrous dichloromethane at room temperature. The solution was cooled to 0 °C and DIPC (0.17 ml,1 mmol), DMAP (122 mg,1 mmol) and camptothecin (348 mg, 1 mmol) were added, in that order, and stirred for 2 h at 0 °C. The reaction mixture was allowed to warm to room temperature and left for 16 h. After filtration, the filtrate was washed with 0.1N HCl, dried (anhydrous MgSO_4_) and evaporated under reduced pressure to yield the crude product as a light yellow solid. An analytical sample of **3a-c** was prepared by flash chromatography (silica; CHCl_3_/MeOH 20:1).

**3a**: light-yellow solid, yieid 82%, IR ν: 3340.7, 2967.3, 2875.3, 1752.9, 1670.8, 1620.3, 1572.9, 1460.4 cm^-1^. ^1^H-NMR (400 MHz, CDCl_3_): δ 8.38 (1H, s, H-7), 8.20 (1H, d, *J* = 8.4Hz, H-12), 7.92 (1H, d, *J* = 8.4Hz, H-9), 7.81 (1H, t, *J* = 7.2Hz, H-10), 7.64 (1H, t, *J* = 7.2Hz, H-11), 7.25 (1H, s, H-14), 5.51 (2H, ABq, *J* = 17.2Hz, H-17), 5.25 (2H, s, H-5), 4.26-4.16 (2H, m), 3.87-3.79 (2H, m), 3.61 (30H, br, mPEG), 3.35 (3H, s, mPEG-OCH_3_), 2.85-2.81 (2H, m, *J* = 7.4Hz, mPEG), 2.66 (2H, t, *J* = 6.8Hz, mPEG), 2.26-2.10 (2H, m, H-19), 0.97 (3H, t, *J* = 7.6Hz, H-18). ^13^C-NMR (100 MHz, CDCl_3_): δ 7.72, 23.63, 28.98, 31.87, 42.09, 50.04, 59.13, 64.11, 67.14, 69.06, 70.64, 72.03, 76.35, 96.35, 120.11, 128.13, 128.27, 128.34, 128.58, 129.72, 130.76, 131.29, 146.02, 146.36, 148.96, 152.48, 157.20, 157.46, 167.51, 171.41, 171.88.

**3b**: light-yellow solid, yield 85%, IR ν: 3445.7, 2941.3, 2872.8, 1753.0, 1672.2, 1623.8, 1562.4, 1458.1 cm^-1^. ^1^H-NMR (400 MHz, CDCl_3_): δ 8.38 (1H, s, H-7), 8.20 (1H, d, *J* = 8.4 Hz, H-12), 7.92 (1H, d, *J* = 8.4 Hz, H-9), 7.81 (1H, t, *J* = 7.2 Hz, H-10), 7.64 (1H, t, *J* = 7.2 Hz, H-11), 7.25 (1H, s, H-14), 5.51 (2H, ABq, *J* = 17.2 Hz, H-17), 5.25 (2H, s, H-5), 4.26-4.16 (2H, m), 3.87-3.79 (2H, m), 3.61 (30H, br, mPEG), 3.35 (3H, s, mPEG-OCH_3_), 2.85-2.81 (2H, m, *J* = 7.4 Hz, mPEG), 2.66 (2H, t, *J* = 6.8 Hz, mPEG), 2.26-2.10 (2H, m, H-19), 0.97 (3H, t, *J* = 7.6 Hz, H-18). ^13^C-NMR (100 MHz, CDCl_3_): δ 7.73, 23.63, 28.98, 31.87, 42.15, 50.04, 59.15, 64.11, 67.14, 69.07, 70.65, 72.04, 76.35, 96.34, 120.14, 128.12, 128.27, 128.34, 128.60, 129.73, 130.75, 131.28, 146.00, 146.36, 148.97, 152.50, 157.11, 157.46, 167.49, 171.41, 171.87.

**3c**: light-yellow solid, yield 78%, IR ν: 3440.1, 2885.7, 1750.0, 1665.8, 1618.5, 1564.8, 1467.9 cm^-1^. ^1^H-NMR (400 MHz, CDCl_3_): δ 8.39 (1H, s, H-7), 8.21 (1H, d, *J* = 8.4 Hz, H-12), 7.93 (1H, d, *J* = 7.2 Hz, H-9), 7.82 (1H, t, *J* = 7.2 Hz, H-10), 7.66 (1H, t, *J* = 7.2Hz, H-11), 5.52 (2H, ABq, *J* = 16.8 Hz, H-17), 5.27 (2H, s, H-5), 4.22 (4H, s), 3.87-3.79 (2H, m), 3.63 (323H, br, mPEG), 3.36 (6H, s, mPEG-OCH_3_), 2.83 (2H, s, mPEG), 2.73-2.66 (4H, m, mPEG), 2.14 (2H, m, H-19), 0.97 (3H, t, *J* = 7.6Hz, H-18). ^13^C-NMR (100 MHz, CDCl_3_): δ 7.68, 23.60, 28.93, 31.81, 42.02, 50.00, 59.11, 64.06, 65.91, 67.08, 69.02, 70.63, 71.99, 76.29, 96.27, 120.08, 128.07, 128.22, 128.30, 128.55, 129.68, 130.70, 131.25, 145.93, 146.31, 148.91, 152.46, 157.40, 167.42, 171.35, 171.80.


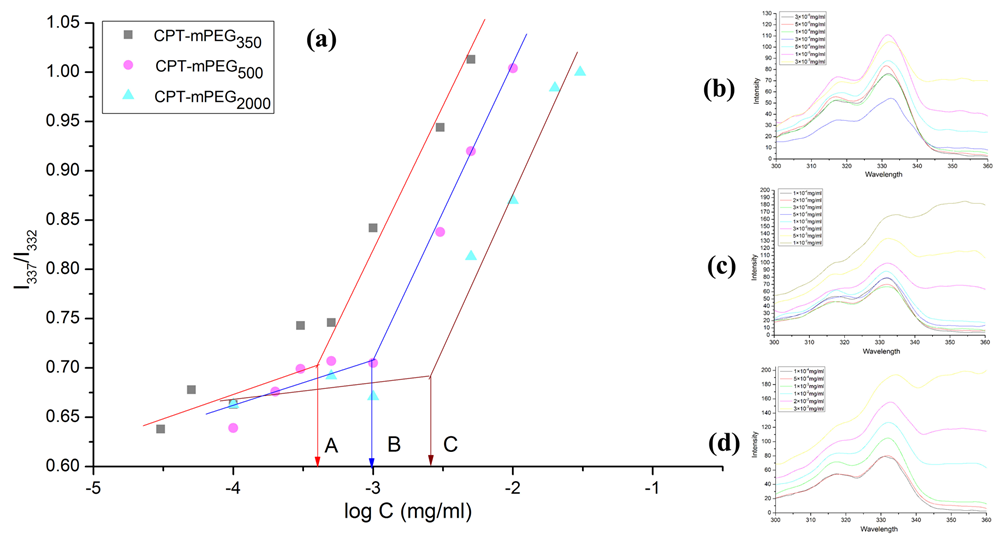


**Fig. S1.** (a) Plot of the intensity ratio I_337_/I_332_ (from pyrene excitation spectra) as a function of log C, and fluorescence spectrum of mPEG_350_-CPT (b), mPEG_500_-CPT (c) and mPEG_2000_-CPT (d).
